# Optimizing Textile Disinfection in Hospital-Associated Infections Using Gaseous Ozone

**DOI:** 10.3390/pathogens14100977

**Published:** 2025-09-26

**Authors:** Francesco De Caro, Federica Dell’Annunziata, Oriana Motta, Nicoletta Capuano, Antonio Faggiano, Leonardo Aulisio, Matteo Tomeo, Emanuela Santoro, Giovanni Boccia, Mario Capunzo, Giuseppina Moccia, Veronica Folliero, Gianluigi Franci

**Affiliations:** 1 Department of Medicine, Surgery and Dentistry, Scuola Medica Salernitana, University of Salerno, 84081 Baronissi, Italy; fdecaro@unisa.it (F.D.C.); federica.dellannunziata@unisa.it (F.D.); omotta@unisa.it (O.M.); niccapuano@unisa.it (N.C.); laulisio@unisa.it (L.A.); mtomeo@unisa.it (M.T.); esantoro@unisa.it (E.S.); gboccia@unisa.it (G.B.); mcapunzo@unisa.it (M.C.); gfranci@unisa.it (G.F.); 2 Public Health Laboratory for the Analysis of Community Health Needs, Department of Medicine and Surgery, University of Salerno, Baronissi Campus, 84081 Baronissi, Italy; 3 Department of Experimental Medicine, University of Campania Luigi Vanvitelli, 80138 Naples, Italy; 4 Department of Chemistry and Biology “Adolfo Zambelli”, University of Salerno, 84084 Fisciano, Italy; anfaggiano@unisa.it; 5 U.O.S. Microbiology and Virology, A.O.U. San Giovanni di Dio e Ruggi d’Aragona, 84131 Salerno, Italy

**Keywords:** healthcare-associated infections, antimicrobial resistance, ozone, disinfection, textile decontamination, work safety

## Abstract

Healthcare-associated infections (HAIs) pose a significant risk in clinical settings by extending hospitalization times and increasing healthcare costs. This study aimed to evaluate the effectiveness of gaseous ozone, generated by an automatic rotary dispenser, in disinfecting hospital fabrics contaminated with common HAI-related pathogens. The antimicrobial efficacy of ozone was tested on cotton, polyester, and blended fabrics artificially contaminated with *Staphylococcus aureus*, *Escherichia coli*, and *Candida albicans*. The fabrics were exposed to ozone treatment cycles of 25 and 45 min. Additional tests were conducted on layered fabrics to assess ozone penetration into folds and seams. A 25 min ozone exposure significantly reduced the microbial load on all tested fabrics. A 45 min cycle resulted in an almost complete elimination of the tested pathogens. Ozone also effectively disinfected inner fabric layers, indicating its ability to reach areas typically resistant to conventional cleaning methods. Gaseous ozone demonstrates high efficacy as a disinfectant for hospital textiles, offering thorough decontamination across various materials and fabric structures. This technology represents a sustainable, residue-free alternative to traditional disinfection methods and promises to reduce the transmission of HAIs in healthcare environments.

## 1. Introduction

Healthcare-associated infections (HAIs) are infections that patients acquire during their stay in hospital or other healthcare facilities, which were not present or incubating at the time of admission [1,2]. HAIs significantly extend the average length of hospital stay (LOS), escalating healthcare costs [3]. According to the European Centre for Disease Prevention and Control (ECDC) in 2023, approximately 4.5 million HAIs occur annually in Europe, with 30% of these cases resulting in patient death [4,5]. The prevalence of HAIs varies globally, with rates around 5% in North America and Europe, and reaching as high as 40% in some Asian, Latin American, and African countries [6,7,8]. The World Health Organization (WHO) classifies the primary pathogens responsible for HAIs into three priority levels: (i) critical, which includes pathogens such as *Acinetobacter baumannii*, Enterobacteriaceae, *Mycobacterium tuberculosis*; (ii) high, encompassing *Salmonella typhi*, *Shigella* spp., *Enterococcus faecium*, *Pseudomonas aeruginosa*, non-typhoidal *Salmonella*, *Neisseria gonorrhoeae*, *Staphylococcus aureus* (*S. aureus*); and (iii) medium, which includes Group A-B Streptococci, *Streptococcus pneumoniae*, *Haemophilus influenzae* [9,10]. Alongside bacterial pathogens, fungi are significant contributors to HAIs, particularly through the contamination of permanent devices. Candida species, including *Candida albicans* (*C. albicans*), *Candida parapsilosis*, and *Candida glabrata*, are among the most common fungal agents associated with HAIs [11]. Managing these infections often requires prolonged antimicrobial therapy, which can lead to adverse effects and increased antimicrobial resistance [11]. The Centers for Disease Control and Prevention (CDC) has identified three main ways that microbes spread in healthcare settings: through air, respiratory droplets and direct or indirect contact, such as hospital textiles or medical devices [12]. Nosocomial infections primarily result from poor implementation and adherence to safety guidelines and protocols in hospital environments, which are inherently non-sterile and where daily practices can increase the risk of pathogen transmission. Effective infection prevention and control require rigorous adherence to cleaning, disinfection, and sterilization standards, along with ongoing staff training and monitored procedures [13,14,15]. Proper hygiene procedures are crucial to avoid contamination of surfaces, reusable medical devices, hospital linens, and healthcare personnel’s work clothing [16]. The U.S. Food and Drug Administration (FDA) emphasizes the essential role of educating healthcare workers on effective cleaning, disinfection, and sterilization practices [17]. Over time, hospital uniform laundering processes have advanced markedly, evolving from traditional steam laundries to advanced high-tech washing machines that facilitate continuous washing [18]. Effective washing procedures to counteract bacterial contamination require a combination of chemical, thermal, and mechanical factors [19]. Recommended practices involve washing at temperatures between 60 and 71 °C, using detergents combined with active oxygen bleach and/or chlorine bleach, and maintaining a cycle duration of 25 min [20]. However, the risk of recontamination must be considered, particularly due to the potential formation of microbial biofilms inside washing machines during the cleaning process [21]. To improve the efficiency of hospital textile cleaning, quaternary ammonium compounds (QACs), such as benzalkonium chloride (BAC) and dimethyl didecyl ammonium chloride (DDAC), are commonly employed [22]. These compounds effectively reduce Gram-positive bacterial load even at lower temperatures. In contrast, Gram-negative bacteria and fungi often require higher concentrations and temperatures for effective eradication [23]. Heat treatments, such as dry steam, are also used, particularly for garments with porous surfaces. However, conventional methods face limitations, especially in thoroughly disinfecting hard-to-reach areas such as folds, lapels, and seams [24,25]. Therefore, there is a pressing need to develop and implement methodologies to enhance disinfection performance. Ozone is emerging as a promising innovation for disinfecting surfaces, sterilizing medical devices, and sanitizing personal protective equipment (PPE) [26,27]. As a potent oxidizing agent, ozone is widely used across various industrial sectors, including food processing, public health, the textile industry, and water treatment. In the textile industry, O_3_ is utilized for the pretreatment of cotton dyeing and the bleaching of textile fibers, demonstrating its versatility and effectiveness [28,29]. Ozone provides a cost-effective and environmentally friendly alternative to traditional disinfectants, decomposing naturally into oxygen without producing harmful byproducts [30]. Its well-documented sporicidal and virucidal properties are effective against a broad spectrum of microorganisms, including viruses, bacteria, and fungi [31]. This versatility makes it an excellent choice for decontamination processes [32]. O_3_ has recently proven effective in healthcare settings against Norovirus and SARS-CoV-2, as well as various multidrug-resistant bacteria and fungi that are resistant to conventional antimicrobials [33,34]. The effectiveness of ozone as a disinfectant is influenced by several factors, including concentration, duration of exposure, temperature, environmental conditions, and pH level [35,36]. Studies have shown that disinfection efficiency improves at temperatures between 5 and 30 °C, accelerating ozone decomposition into molecular oxygen [37]. Consistent ozone concentration throughout sterilization ensures a controlled environment that promotes effective microbial inactivation. Adjusting ozone concentration and exposure time are key factors for optimizing the productivity and efficiency of decontamination cycles [35,38]. The use of ozone has also been extended to the decontamination of hospital textiles. Ozonated water washing systems have established themselves as an alternative to conventional cycles, enabling high-level decontamination of infected linens with reduced use of chemical detergents and a significant reduction in energy consumption [39].

A dedicated ozone dispenser, like the Rotary Automatic Distributor 900, represents a breakthrough in fabric sanitization, enabling effective decontamination without high water consumption, detergents, or high temperatures. Unlike traditional washing methods, this innovative technology ensures deep ozone penetration into folds and multilayered materials, reaching areas that are typically inaccessible. The device features an advanced sanitization system with two exposure programs, offering flexible treatment cycles of 25 and 45 min. Tests have been conducted on healthcare workers’ uniforms made of various fabrics, including cotton, synthetic, and semi-synthetic materials, demonstrating the device’s superior efficacy against Gram-positive and Gram-negative bacteria, as well as yeasts. Furthermore, the controlled diffusion of ozone has been carefully evaluated to minimize worker exposure while maintaining high safety standards, combining innovation, ease of use, and environmental sustainability.

## 2. Materials and Methods

### 2.1. Rotating Dispenser

The currently commercially available 900 rotary vending machine (Zucchetti Centro Sistemi, Arezzo, Italy) used in this study [40]. The cabinet enables the decontamination of tools, personal protective equipment (PPE), instruments, garments, and textiles, particularly where treatments involving heat, chemical agents, or UV light are not feasible. This cabinet is ideal for schools, libraries, work and storage areas, medical and dental offices, shops, hotels, and more. Key features include:

- Fully Hermetic Design: Ensures an airtight environment for effective decontamination cycles.
- Catalyst System: Equipped with a catalyst to accelerate ozone conversion back into oxygen, thus shortening treatment cycles and ensuring safe post-treatment conditions.
- Adjustable Shelving.

**Figure 1:**
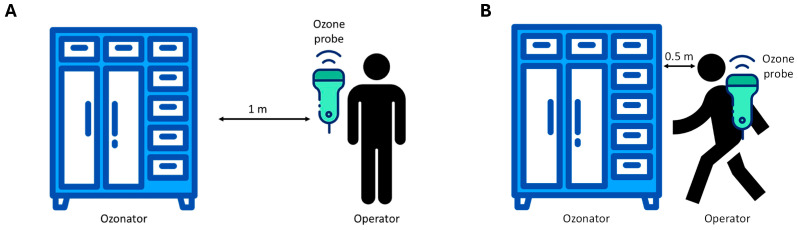
Experimental scheme of the ozone exposure test at 1 m (**A**) and 50 cm (**B**) distance of the operator from the ozonizer.

To ensure the correct use of ozone as a disinfectant agent and guarantee the safety of operators in the workplace, the potential ozone diffusion around the device was monitored through specific tests designed to measure ozone dispersion during a standard ozonization cycle. A GrayWolf DirectSense IQ-610 probe was utilized to monitor ozone concentration [36]. The device operated within a non-condensing environment with an average temperature of 20 °C. Measurements were logged and analyzed using GrayWolf’s WolfSense v6.3.2 software, ensuring high accuracy and reliability in indoor air quality assessment data collection. The detection device was positioned at an average human height (1.70 m), testing effectiveness at two distances from the ozonizer. In the first set of tests, the measurement device was placed 1 m away from the ozonizer. This setup aimed to simulate the potential exposure of a worker approaching the device during daily routines, as illustrated in Figure 1A. An exposure time of 1.5 h near the ozonizer was considered to represent a limit scenario. Subsequently, a second set of tests was conducted with the detector placed approximately 50 cm from the ozonizer (Figure 1B). This series of measurements evaluated the ozone exposure a worker might experience when using the ozonizer to sterilize work uniforms. Similarly, an exposure duration of 1.5 h was considered for this scenario. This methodological approach allowed for a comprehensive evaluation of ozone exposure under realistic occupational conditions. Measurements were continuously recorded for a total of 8 h, covering both the ozonization phase (1.5 h) and the subsequent decay of residual ozone. Time-weighted average (TWA) values were then calculated over these 8 h, ensuring comparability with established occupational exposure limits.

### 2.2. Bacterial Strains and Growth Conditions

Antimicrobial assays were conducted using the following strains: *S. aureus* purchased from American Type Culture Collection (ATCC 6538, Manassan, VA, USA), Gram-positive bacteria, nonsporulating, aerobic and anaerobic optional, immobile and belonging to the Staphylococaceae family; *Escherichia coli* (*E. coli*) (ATCC 25922, Manassan, VA, USA), Gram-negative bacteria, nonsporulating, aerobic and anaerobic optional, mobile and belonging to the Enterobacteriaceae family; *C. albicans* (ATCC 10231, Manassan, VA, USA), yeasts, nonsporulating, aerobic and anaerobic optional, immobile and belonging to the family of Debaryomycetaceae. All strains were seeded on Brain Heart Infusion (BHI) agar plates (Sigma-Aldrich, St. Louis, MO, USA), overnight (ON), at 37 °C; subsequently, fresh colonies were inoculated into liquid BHI for pre-inoculum and incubated ON, at 37 °C, under orbital shaking (180 revolutions per minute). Then, a fresh inoculum for each strain was prepared until it reached half the logarithmic exponential phase (optical density, OD = 0.5, 10^7^–10^9^ CFU/mL). Serial dilutions were prepared to achieve the bacterial concentration required for the test (10^4^–10^5^ CFU/mL) [41,42].

### 2.3. Antimicrobial Activity Tests

The effectiveness of reducing the microbial load was evaluated using a 24 Volt compact rotating dispenser with a 10″ display (Zucchetti Centro Sistemi, Arezzo, Italy). Plates seeded with *E. coli*, *S. aureus*, and *C. albicans* were placed in the dispenser according to exposure programs 1 and 2 (25 and 45 min, ozone concentration 4.5 ppm). The same plates were incubated at room temperature (RT) for the entire test duration and used as growth control. To account for potential environmental contamination (positive control, CTRL+), sterile BHI agar plates were positioned both inside and outside the dispenser and exposed for the same duration to ozone. Then, all plates were incubated at 37 °C to promote microbial growth. On the next day, the colonies were counted and converted to Colony Forming Units (CFU) per mL (CFU/mL). All experiments were conducted under controlled conditions within the ozone dispenser’s chamber, with the temperature set at 18–19 °C and the relative humidity maintained between 45% and 55%, as specified by the manufacturer for optimal disinfection performance.

### 2.4. Tests on Textile Materials

**Figure 2:**
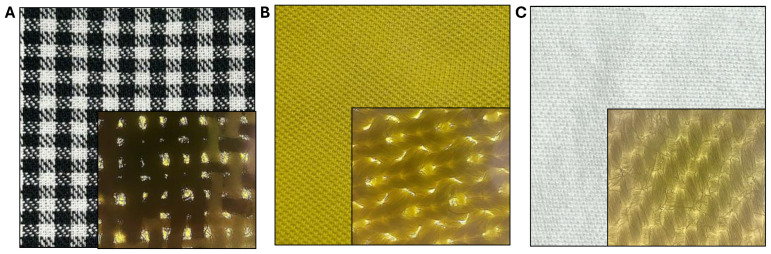
Hospital clothing used in this study, composed of 100% cotton (**A**), 100% polyester (**B**) and a 40% cotton–60% polyester blend (**C**); for each, an area of 25 cm^2^ was sectioned and contaminated with *S. aureus*, *E. coli*, and *C. albicans*, respectively.

The antimicrobial efficacy of ozone was assessed on various types of hospital garments artificially contaminated with *Staphylococcus aureus*, *Escherichia coli*, and *Candida albicans*. For this purpose, three fabric types were selected: 100% cotton, 100% polyester, and a 40% cotton–60% polyester blend. From each fabric, a 25 cm^2^ area was isolated for the experimental assays (Table 1, Figure 2). The hospital clothing was subjected to sterilization by UV radiation for 40 min. Subsequently, sterile tissues were contaminated with 100 µL (10^4^–10^5^ CFU/mL) of fresh inoculum. To ensure uniform coverage of the entire surface, the total volume was applied to 25 cm^2^ fabric samples, divided into five 20 µL aliquots. The samples were subsequently exposed in the 24-volt compact rotary dispenser for 25 and 45 min cycles (programs 1 and 2) at an ozone concentration of 4.5 ppm. The same tissues were used as growth controls outside the instrument, under the same contamination conditions and exposure times. After treatment, the fabrics were immersed in 10 mL of Phosphate-Buffered Saline (PBS) 1× and seeded on BHI agar plates at serial dilutions (10^−1^, 10^−2^, 10^−3^, 10^−4^, and 10^−5^) to observe the microbial growth. The plates were incubated for 24–48 h at 37 °C and counted to define the CFU/mL. Additional tests evaluating the effectiveness of ozone at inhibiting microbial growth were conducted under the same contamination conditions and exposure times, utilizing only the instrument’s ventilation system. The capacity of ozone to penetrate through multiple fabric layers was evaluated. Five layers (25 cm^2^ each) of a fabric composed of 40% cotton and 60% polyester were individually inoculated with *S. aureus*, *E. coli*, and *C. albicans*. The layers were then stacked in sequence and subjected to an ozonation cycle over a period of 45 min. Again, after ozonation, each tissue sample was aseptically transferred to 10 mL of sterile 1× PBS and homogenized by vortexing. Tenfold serial dilutions (from 10^−1^ to 10^−5^) were prepared, and aliquots (100 µL) of each dilution were seeded onto BHI plates. After incubation at 37 °C for 24–48 h, colonies were enumerated as CFU/mL to assess the number of viable microorganisms.

### 2.5. Statistical Analysis

Statistical analysis was performed using GraphPad Prism 9.0 software (San Diego, CA, USA). Data are presented as mean ± standard deviation (SD) from three independent experiments. The significance of the difference between samples treated and not treated with ozone was obtained using a Student’s *t*-test. The *p*-value < 0.05 was considered significant.

## 3. Results

### 3.1. Work Scenario and Safety

**Figure 3:**
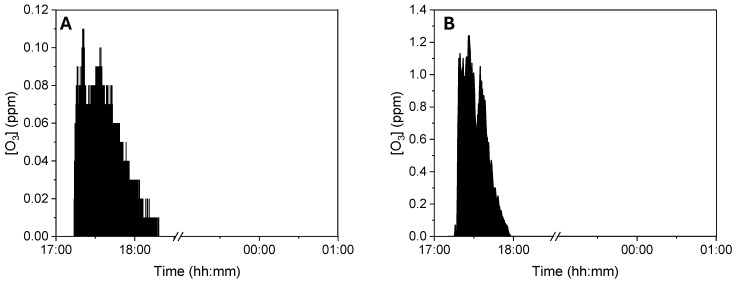
Ozone TWA analysis measured 1 m (**A**) and 50 cm (**B**).

The first scenario examined ozone exposure for a worker passing near the ozonizer during regular work activities. The time-weighted average (TWA) ozone concentration measured at this 1 m distance was found to be below 0.1 ppm. This TWA value, in compliance with the Occupational Safety and Health Administration (OSHA) exposure limit of 0.1 ppm, indicates that the working environment adheres to current safety regulations, ensuring health protection for workers exposed to potentially harmful substances like ozone (Figure 3A). The second scenario investigated ozone exposure for a worker using the ozonizer to sterilize work uniforms, with measurements taken at approximately 50 cm from the device. Although the peak concentrations measured 50 cm from the device were significantly higher, the calculated TWA (time-weighted average) also remained below 0.1 ppm due to the short duration of these peaks and the overall exposure pattern (Figure 3B). This specific TWA value demonstrates compliance with OSHA occupational exposure limits, confirming that the safety measures in place effectively restrict ozone exposure to safe levels, even close to the ozonizer. This result highlights the device’s design, which effectively addresses ozone-related health risks, thereby ensuring the safety of workers.

### 3.2. Ozone Activity Against Bacteria and Fungi

**Figure 4:**
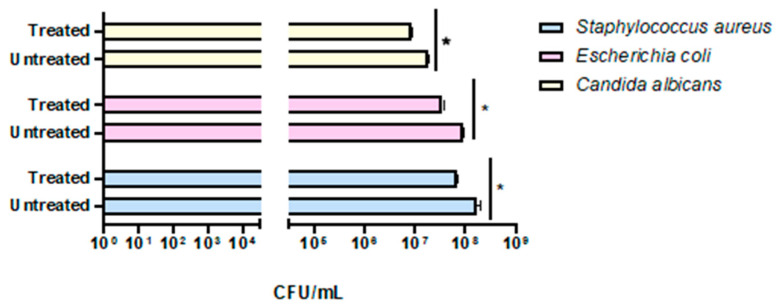
Evaluation of the effectiveness of the compact rotary ozone distributor on plates seeded with *E. coli*, *S. aureus*, and *C. albicans*. Program 1 (25 min). Summary histogram of colonies counted and converted to CFU/mL of *S. aureus*, *E. coli*, and *C. candida* treated and untreated with ozone. Data represent the mean ± standard deviation (SD) of three independent experiments. *: *p*-value < 0.035.

**Figure 5:**
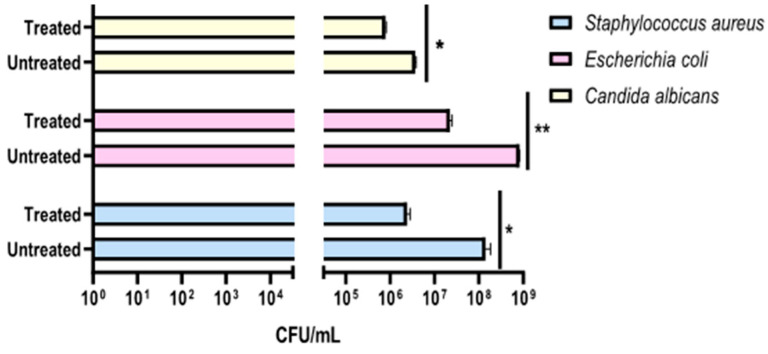
Evaluation of the effectiveness of the compact rotary ozone distributor on plates seeded with *E. coli*, *S. aureus*, and *C. albicans*. Program 2 (45 min). Summary histogram of colonies counted and converted to CFU/mL of *S. aureus*, *E. coli*, and *C. candida* treated and untreated with ozone. Data represent the mean ± standard deviation (SD) of three independent experiments. *: *p*-value < 0.04; **: *p*-value = 0.0075.

The preliminary effectiveness of a compact 24 Volt rotary dispenser with a 10″ display was evaluated by exposing plates seeded with *E. coli*, *S. aureus* and *C. albicans* to 4.5 ppm of ozone for 25 and 45 min. Bacterial load reduction was evaluated relative to the growth control, consisting of identically contaminated plates maintained at room temperature throughout the test Furthermore, sterile BHI-agar plates were inserted inside and outside the dispenser to confirm the absence of environmental contaminants, representing the CTRL+. The obtained results recorded that the exposure to 4.5 ppm of ozone for 25 min (program 1) significantly reduced the microbial load of all strains, compared to untreated ones (Figure 4). In detail, a 0.38-log reduction (*p*-value = 0.04) for *S. aureus* was encountered, with a microbial decrease from 1.5 × 10^8^ CFU/mL (Figure S1A) to 6.3 × 10^7^ CFU/mL (Figure S1B). Similarly, the *E. coli* load decreased 0.38-log (*p*-value = 0.06), recording 8.8 × 10^7^ CFU/mL in the untreated sample (Figure S1C) and 3.7 × 10^7^ CFU/mL after ozone exposure (Figure S1D). Regarding antifungal activity, a comparable effect was verified, detecting a reduction of 0.34 log (*p*-value = 0.03) for *C. albicans*, from 1.9 × 10^7^ CFU/mL in the untreated sample (Figure S1E) to 8.8 × 10^6^ CFU/mL in treated ones, respectively (Figure S1F). The second test involved ozone exposure for a longer time (45 min, ozone concentration 4.5 ppm), resulting in a greater reduction in the tested loads (Figure 5). Indeed, a time exposure of almost double the previous one reduced the *S. aureus* load by 1.75 times (*p*-value = 0.002), with a recorded value of 1.1 × 10^8^ CFU/mL in the untreated sample (Figure S2A) compared to 2.0 × 10^6^ CFU/mL in the treated sample (Figure S2B). Also, for *E. coli*, a reduction rate of 1.61 log (*p*-value = 0.04) was found, with 8.2 × 10^8^ CFU/mL without ozone exposure (Figure S2C) and 2.0 × 10^7^ CFU/mL after treatment (Figure S2D). On the other hand, for *C. albicans*, ozone prolonged exposure determined a 0.65-log reduction in colonies (*p*-value *=* 0.05), higher than the previous cycle, from 3.6 × 10^6^ CFU/mL (Figure S2E) to 8.0 × 10^5^ CFU/mL (Figure S2F). Table 2 summarizes the bacterial and fungal load trends in response to the different ozone exposure programs.

### 3.3. Exposure of Hospital Garments to Ozone

**Figure 6:**
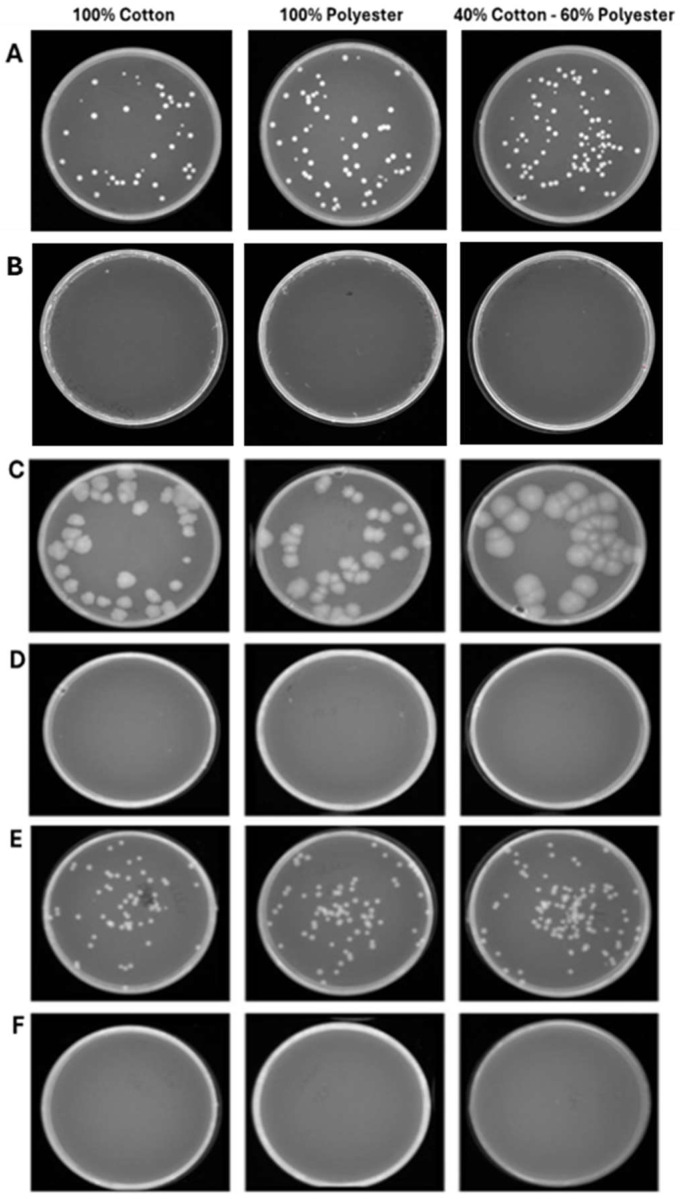
Program 1 (25 min): microbial growth from untreated (**A**) and treated (**B**) *S. aureus* contaminated-fabrics; untreated (**C**) and treated (**D**) *E. coli*-contaminated fabrics; untreated (**E**) and treated (**F**) *C. albicans*-contaminated fabrics. Data represent three independent experiments’ mean ± standard deviation (SD).

**Figure 7:**
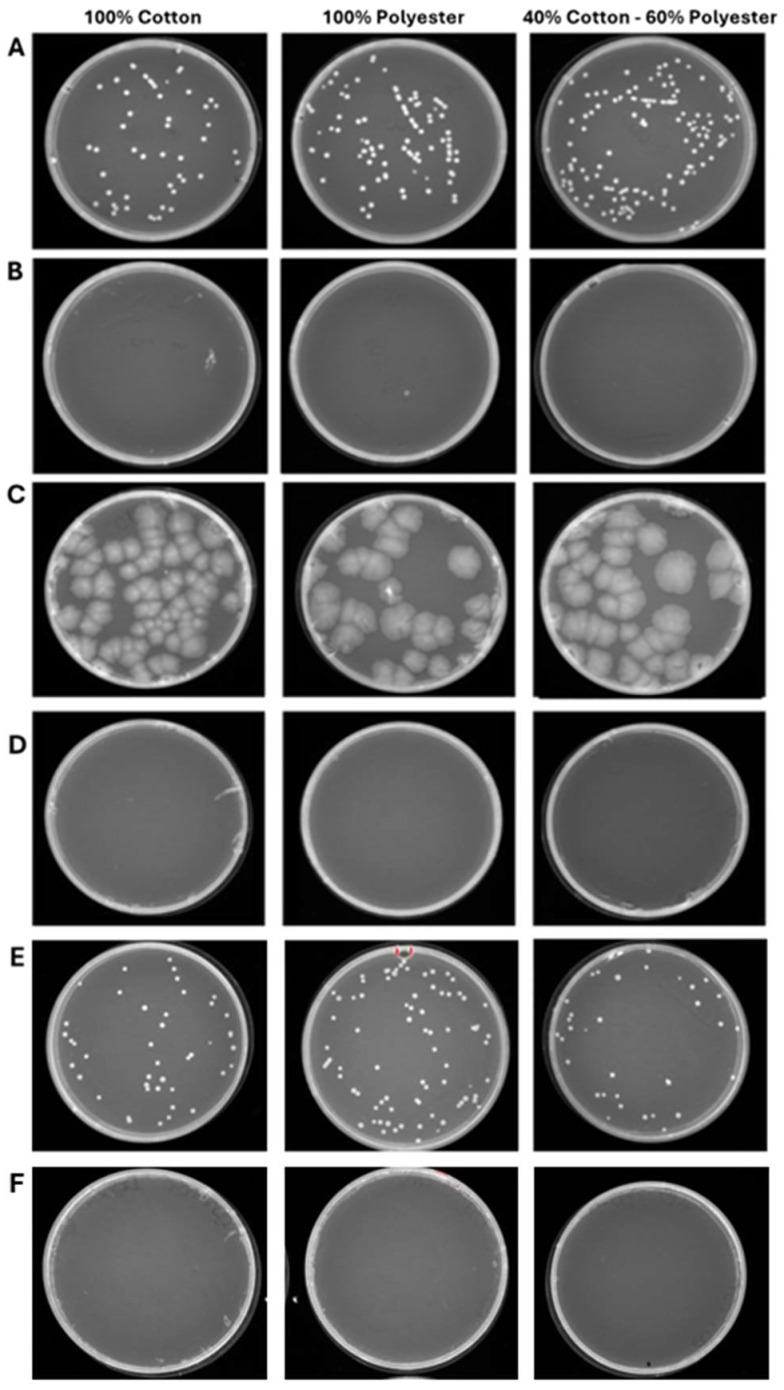
Program 2 (45 min): microbial growth from untreated (**A**) and treated (**B**) *S. aureus*-contaminated fabrics; untreated (**C**) and treated (**D**) *E. coli*-contaminated fabrics; untreated (**E**) and treated (**F**) *C. albicans*-contaminated fabrics. Data represent three independent experiments’ mean ± standard deviation (SD).

The second experimental phase involved evaluating the antimicrobial efficacy of ozone in healthcare textiles with varying compositions sourced from the hospital setting. Therefore, three fabrics (100% cotton, 100% polyester and 40% cotton–60% polyester) were selected and contaminated with *S. aureus*, *E. coli*, and *C. albicans*, at 10^4^–10^5^ CFU/mL loads. After, the ozonation cycles of 25 and 45 min were conducted to evaluate the microbial reduction. The results clearly showed that a total reduction in the bacterial and fungal load was already verified after 25 min (Figure 6). Indeed, in the 100% cotton, 100% polyester, and 40% cotton–60% polyester controls the contamination load was, respectively, 2.7 × 10^4^–2.0 × 10^4^–1.3 × 10^4^ CFU/mL for *S. aureus* (Figure 6A), 1.4 × 10^4^–1.4 × 10^4^–1.3 × 10^4^ CFU/mL for *E. coli* (Figure 6C), and 2.0 × 10^4^–2.6 × 10^4^–3.0 × 10^4^ CFU/mL for *C. albicans* (Figure 6E). On the other hand, 25 min ozonation cycle was sufficient to completely reduce the microbial load, confirmed by the absence of colonies after 48 h of incubation at 37 °C. A similar trend was maintained for longer exposure times (45 min) in which the contamination of 100% cotton, 100% polyester and 40% cotton–60% polyester healthcare textiles were, respectively, 1.8 × 10^4^–1.9 × 10^4^–1.3 × 10^4^ CFU/mL for *S. aureus* (Figure 7A), 2.0 × 10^4^–1.4 × 10^4^–1.3 × 10^4^ CFU/mL for *E. coli* (Figure 7C), and 2.0 × 10^4^–2.5 × 10^4^–3.3 × 10^4^ CFU/mL for *C. albicans* (Figure 7E). No microbial growth was observed following ozone exposure. Table 3 and Table 4 summarize the differences in bacterial/fungal load, expressed as log reduction, of each type of garment exposed and not exposed to ozone.

**Figure 8:**
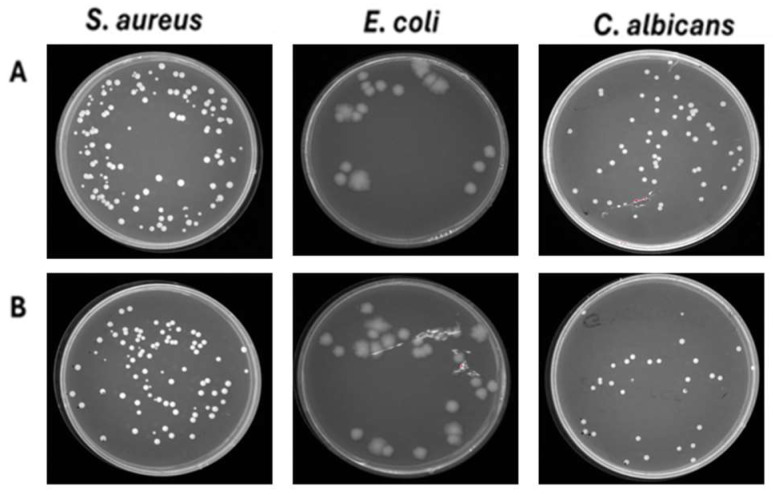
Effect of ventilation against *S. aureus, E. coli and C. albicans* deposited on 40% cotton–60% polyester fabrics. (**A**) Untreated contaminated fabrics. (**B**) Treated contaminated fabrics.

**Figure 9:**
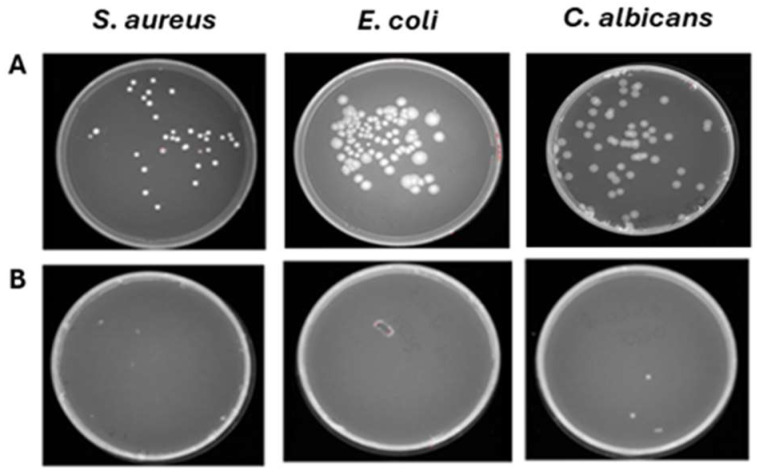
Effect of ozone against *S. aureus*, *E. coli*, and *C. albicans* deposited on 5 layers of 40% cotton–60% polyester fabric. (**A**) Untreated contaminated fabrics. (**B**) Treated contaminated fabrics.

To further confirm that the reduction in microbial load was attributable to ozone, tests were conducted on the mixed-composition fabric (40% cotton, 60% polyester) contaminated with *S. aureus*, *E. coli*, and *C. albicans*, which was subjected to a 45 min ventilation-only cycle. The results found confirmed that ventilation did not induce a significant difference, recording approximately the same number of colonies (8.1 × 10^4^ CFU/mL for *S. aureus*, 2.0 × 10^4^ CFU/mL for *E. coli* and 3.2 × 10^4^ CFU/mL for *C. albicans*) compared to the growth control not exposed (Figure 8). To evaluate the ozone’s ability to penetrate deeper through different layers of fabric, an experiment was conducted by placing 5 layers of 40% cotton–60% polyester blend fabric contaminated with *S. aureus*, *E. coli*, and *C. albicans*. The results clearly demonstrate that ozone can penetrate multiple layers of fabric, as evidenced by the absence of residual contamination in any of the tested layers. The complete eradication of *S. aureus* and *E. coli* confirms not only the antimicrobial efficacy of ozone but also its ability to spread uniformly across overlapping textile barriers. In contrast, *C. albicans* showed a distinct response, with ozone exposure leading to a substantial reduction of approximately 1.4 log (from 3.2 × 10^5^ CFU/mL in untreated controls to 4 × 10^3^ CFU/mL in treated samples), although complete eradication was not achieved. This result suggests that the reduced effect observed on fungal cells is not related to a limitation of ozone penetrance, but rather to a lower intrinsic susceptibility of *C. albicans* compared to bacteria. Overall, these results highlight the strong ability of ozone to permeate tissue layers and exert differential antimicrobial action depending on the structural and physiological characteristics of the target microorganisms (Figure 9).

## 4. Discussion

Nosocomial infections represent a critical and complex issue in the healthcare field [43]. The pathogen’s ability to survive on surfaces and hospital clothes increases the risk of cross-contamination between patients and healthcare workers [44]. Traditional disinfection methods do not guarantee complete microbial eradication, resulting in bacteria, viruses and fungi circulation [45]. Hospital textile disinfection procedures are defined by national and international regulations, such as those provided by the Italian National Institute of Health (ISS) and the European standard EN 14065, which specifies the biocontamination control system for laundered textiles [46]. These require the use of high-temperature washing, chemical treatments with disinfectants (e.g., chlorine-based compounds or quaternary ammonium compounds), and regular monitoring of microbial load [18]. However, the effectiveness of these procedures can vary depending on the type of fabric, the level of soiling, and the operating context, and a standardized and reproducible method for assessing their effectiveness on different materials is still lacking [47]. Current guidelines, such as Circular No. 5443 from the Ministry of Health on the management of linens used by COVID-19 patients, offer practical examples of sanitization protocols. However, these recommendations are limited to specific contexts and do not account for variations in fabric types or the reproducibility of treatments. In general, an effective textile treatment should ensure fiber and dye compatibility even after repeated applications, rapid action, thorough penetration through seams, folds, and varying fabric thicknesses, safety for operators, users, and the environment, as well as cost-effectiveness in terms of equipment, installation, and operational use. The document defines that textiles should be collected in marked bags and handled carefully to avoid dust dispersion and contamination of surfaces or people and washed preferably at high temperatures (≥70 °C) with conventional detergents (ISS COVID-19 Report No. 25/2020). These guidelines also highlight conventional methods for monitoring textile disinfection, which typically involve bioburden testing on agar plates or swab sampling. Our study addresses these gaps by evaluating ozone treatment as an alternative or complementary method, using controlled conditions, defined microbial strains, and different fabric types. Ozonation is established as one of the most effective approaches to counteract the spread of hospital pathogens, contributing significantly to reducing HAI. Ozone is a potent oxidizing agent capable of penetrating deeply into textiles, including hard-to-reach regions such as folds, seams, and pockets. Compared to conventional methods, ozone offers several advantages: (i) leaves no toxic residue, (ii) reduces disinfection times, and (iii) does not require the use of chemical agents [48]. Furthermore, it is versatile and can be used on a wide range of fabrics without compromising their integrity [49]. Few studies have used ozone for textile sanitization. Indeed, Pušić et al. used ozone in contaminated textile washing systems, evaluating its sanitization effect by varying the concentrations of detergent, bleach, and ozone at temperatures between 30 °C and 90 °C. The results showed that high concentrations of detergent and bleach ensured the best sanitization, while ozone had a significant effect primarily at low detergent and bleach concentrations and at lower temperatures (30–40 °C), resulting in energy savings. However, ozone production is energy-intensive and, under these conditions, did not significantly improve sanitization compared to conventional processes [39]. A key advantage of using a dedicated ozone dispenser for fabric disinfection, compared to traditional ozonated water washing systems, is the ability to sanitize garments without the need for high water and detergent consumption or high wash temperatures. Although ozonated water systems are effective at reducing microbial load during washing, they still rely on conventional washing infrastructure and are associated with potential recontamination risks during rinsing and handling [50]. In contrast, a stand-alone ozone dispenser, such as the 900 Automatic Rotary Dispenser, allows for drying-cycle decontamination directly on hospital uniforms and textiles. The application of gaseous ozone provides more uniform and thorough sanitization of fabrics, achieving effective penetration into folds, seams, and multilayered materials—areas that are often less accessible with conventional washing methods. Furthermore, gaseous ozone reduces resource consumption and minimizes the risk of cross-contamination, while aqueous ozone is only effective under specific conditions and often requires higher temperatures or combinations with detergents and bleach to achieve comparable results [51]. The closed-cabinet system further enhances safety by offering programmable exposure cycles and on-demand decontamination within the hospital environment, providing flexibility and improved infection control practices. In this context, this study aims to evaluate the effectiveness of ozonation in reducing microbial contamination, using the Automatic Rotating Dispenser 900. The ozone device was evaluated for fabric disinfection using 25 and 45 min cycles. Antimicrobial activity was tested against *S. aureus*, *E. coli*, and *C. albicans* inoculated on cotton, polyester, and blended fabrics. It (4.5 ppm; 25–45 min cycles) demonstrated complete antimicrobial activity against *S. aureus* and *E. coli* and strong activity against *C. albicans* on cotton, polyester, and blended fabrics. Ozone effectively penetrated multiple layers of fabric, achieving total bacterial eradication and a 1.4 log reduction in *C. albicans*. Follow-up testing confirmed that microbial inactivation was attributable solely to ozone exposure, supporting its potential as a versatile disinfection method for healthcare textiles. The present findings were consistent with the other evidence, documenting the ozone’s effectiveness in reducing microbial loads, particularly in the healthcare field. Epelle and colleagues explored the effects of different ozone concentrations and treatment durations on microbial inactivation. The evidence showed over 99% reduction for *S. aureus* at 2 ppm, while higher ozone concentrations (10 ppm) significantly eliminated the *E. coli* load. Regarding fungal species such as *C. albicans* and *Aspergillus fumigatus*, the results indicated greater ozone resistance than bacteria. For *A. fumigatus,* a 30% reduction to 2 ppm was recorded in 16 min, while *C. albicans* showed complete susceptibility. At 20 ppm, both fungi were effectively inhibited after 4 min of exposure. The same study also evaluated real-world scenarios by testing the ozone efficacy on contaminated garments in industrial settings. Consistently, ozone was effective in decontaminating fabrics, although the removal of fungi was slightly less efficient [52]. The same research group provided further insights into ozone disinfection of garments under different operating conditions. Using an ozone chamber, it was evident that dosages of 57 and 83 g·min/m^3^ were effective for textile disinfection, providing several considerations: (i) tightly woven fabrics showed a 43% reduction in ozone penetration compared to looser fabrics; (ii) densely packed garments reduced ozone penetration by 44%, with implications for disinfection efficiency; and (iii) fully stretched trousers showed 14% greater ozone penetration compared to folded ones, suggesting the importance of orientation within the chamber [35]. The overall results highlight ozonation as an effective and safe solution for disinfecting hospital fabrics, interrupting microbial spread and the consequent development of nosocomial infections. The broad penetration and microbial eradication capabilities represent a crucial advantage over traditional disinfection methods. However, further research will be needed to better understand the antimicrobial impact on different types of fabrics and the influence of environmental factors to optimize the disinfection protocol.

## 5. Conclusions

This study demonstrates the benefits of gaseous ozone as a disinfectant for hospital textiles. While previous research, focused primarily on ozonated water systems, highlighting their limited effectiveness unless combined with detergents, bleach, and high temperatures, our results highlight the superior performance of gaseous ozone delivered via a dedicated dispenser [39]. Ensuring uniform penetration into folds, seams, and multilayered fabrics, the 900 automatic rotary dispenser overcomes many of the shortcomings of aqueous ozonation, providing effective microbial eradication without excessive water, chemical, or energy consumption. The results obtained against clinically relevant pathogens such as *S. aureus*, *E. coli*, and *C. albicans* further confirm the versatility and robustness of ozone on a variety of textile types. In line with the findings of Epelle et al. [52], this work reinforces the role of ozone as a promising, environmentally sustainable, and safe disinfection technology in healthcare settings. To date, few studies have used gaseous ozone in closed systems for the disinfection of contaminated textiles. Our results help fill this gap, demonstrating how a dedicated system not only overcomes the limitations of traditional ozonated water washing but also achieves high efficacy with reduced resource consumption and risk of recontamination. The evidence generated provides a basis for further investigation into the potential use of gaseous ozone for managing hospital uniforms and textiles, which could contribute to strengthening overall infection control practices.

## Figures and Tables

**Table 1:** Different compositions of hospital clothing were used in this study.

Tissues	Area (cm^2^)	Materials
Female trousers	25	100% cotton
Male T-shirt	25	100% polyester
Male trousers	25	60% polyester and 40% cotton

**Table 2:** Reduction in microbial load following ozone exposure across different treatment programs.

	Reduction in Microbial Load After Ozone Exposure (Log Reduction)	
Microorganism	Program 1	Program 3
*Staphylococcus aureus*	0.38	1.74
*Escherichia coli*	0.38	1.6141
*Candida albicans*	0.34	0.6545

**Table 3:** Microbial log reduction between tissues treated and untreated with ozone according to Program 1 (25 min).

Reduction in Microbial Load After Ozone Exposure (Log Reduction) Program 1			
	100% Cotton	100% Polyester	40% Cotton–60% Polyester
*S. aureus*	>4.43	>4.30	>4.11
*E. coli*	>4.15	>4.15	>4.11
*C. albicans*	>4.30	>4.41	>4.47

**Table 4:** Microbial log reduction between tissues treated and untreated with ozone according to Program 2 (45 min).

Reduction in Microbial Load After Ozone Exposure (Log Reduction) Program 2			
	100% Cotton	100% Polyester	40% Cotton–60% Polyester
*S. aureus*	>4.25	>4.28	>4.11
*E. coli*	>4.30	>4.15	>4.11
*C. albicans*	>4.30	>4.40	>4.52

## Data Availability

The data presented in this study are available on request from the corresponding author.

## Supplementary Materials

The following supporting information can be downloaded at: https://www.mdpi.com/article/10.3390/pathogens14100977/s1, Figure S1: Evaluation of the effectiveness of the compact ozone rotary dispenser on seeded plates with *E. coli*, *S. aureus*, and *C. albicans*. Program 1 (25 min): microbial growth of untreated *S. aureus* (A) and treated with ozone (B); *E. coli* untreated (C) and treated (D); *C. albicans* untreated (E) and treated (F); Figure S2: Evaluation of the effectiveness of the compact ozone rotary dispenser on seeded plates with *S. aureus*, *E. coli*, and *C. albicans*. Program 2 (45 min): microbial growth of untreated *S. aureus* (A) and treated with ozone (B); *E. coli* untreated (C) and treated (D); *C. albicans* untreated (E) and treated (F).
