# Correction: De Caro et al. Optimizing Textile Disinfection in Hospital-Associated Infections Using Gaseous Ozone. *Pathogens* 2025, *14*, 977

**DOI:** 10.3390/pathogens15030252

**Published:** 2026-02-27

**Authors:** Francesco De Caro, Federica Dell’Annunziata, Oriana Motta, Nicoletta Capuano, Antonio Faggiano, Leonardo Aulisio, Matteo Tomeo, Emanuela Santoro, Giovanni Boccia, Mario Capunzo, Giuseppina Moccia, Veronica Folliero, Gianluigi Franci

**Affiliations:** 1 Department of Medicine, Surgery and Dentistry, Scuola Medica Salernitana, University of Salerno, 84081 Baronissi, Italy; 2 Public Health Laboratory for the Analysis of Community Health Needs, Department of Medicine and Surgery, University of Salerno, Baronissi Campus, 84081 Baronissi, Italy; 3 Department of Experimental Medicine, University of Campania Luigi Vanvitelli, 80138 Naples, Italy; 4 Department of Chemistry and Biology “Adolfo Zambelli”, University of Salerno, 84084 Fisciano, Italy; 5 U.O.S. Microbiology and Virology, A.O.U. San Giovanni di Dio e Ruggi d’Aragona, 84131 Salerno, Italy

## Error in Figure

In the original publication [1], errors were present in Figures 6–9. These errors were due to inaccuracies introduced during the assembly of the figure panels, particularly the accidental overlapping of the plate panels caused by typographical errors in the reconstruction process. 

**Figure 6:**
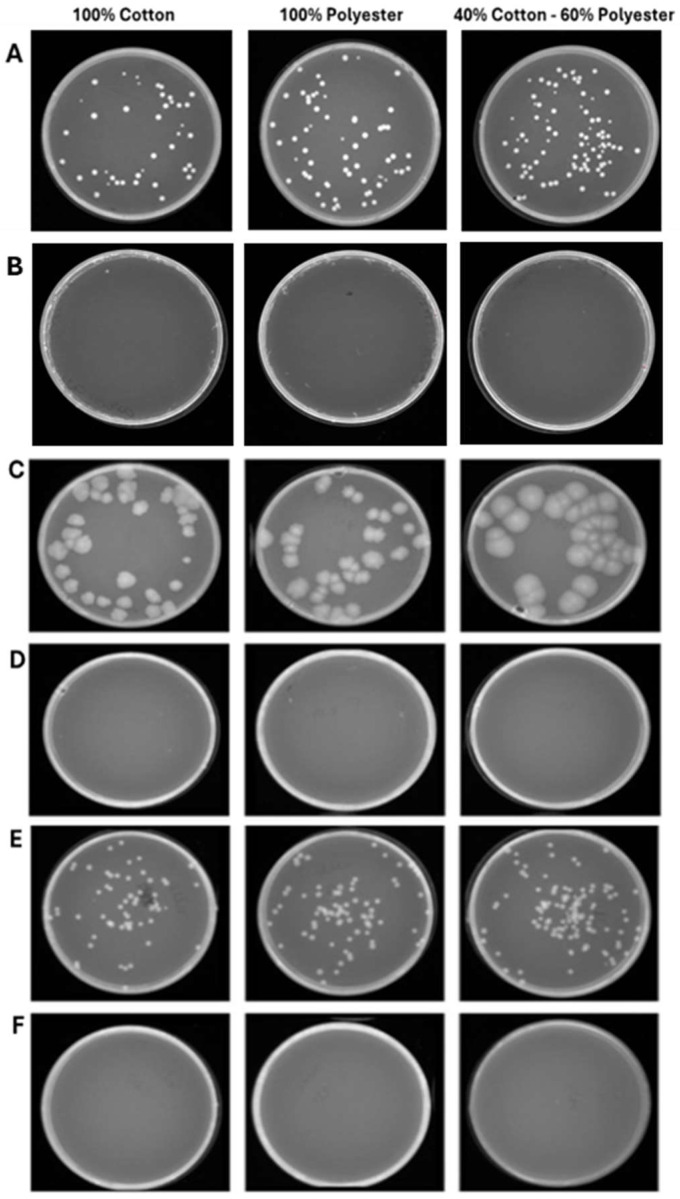
Program 1 (25 min): microbial growth from untreated (**A**) and treated (**B**) *S. aureus* contaminated-fabrics; untreated (**C**) and treated (**D**) *E. coli*-contaminated fabrics; untreated (**E**) and treated (**F**) *C. albicans*-contaminated fabrics. Data represent three independent experiments’ mean ± standard deviation (SD).

**Figure 7:**
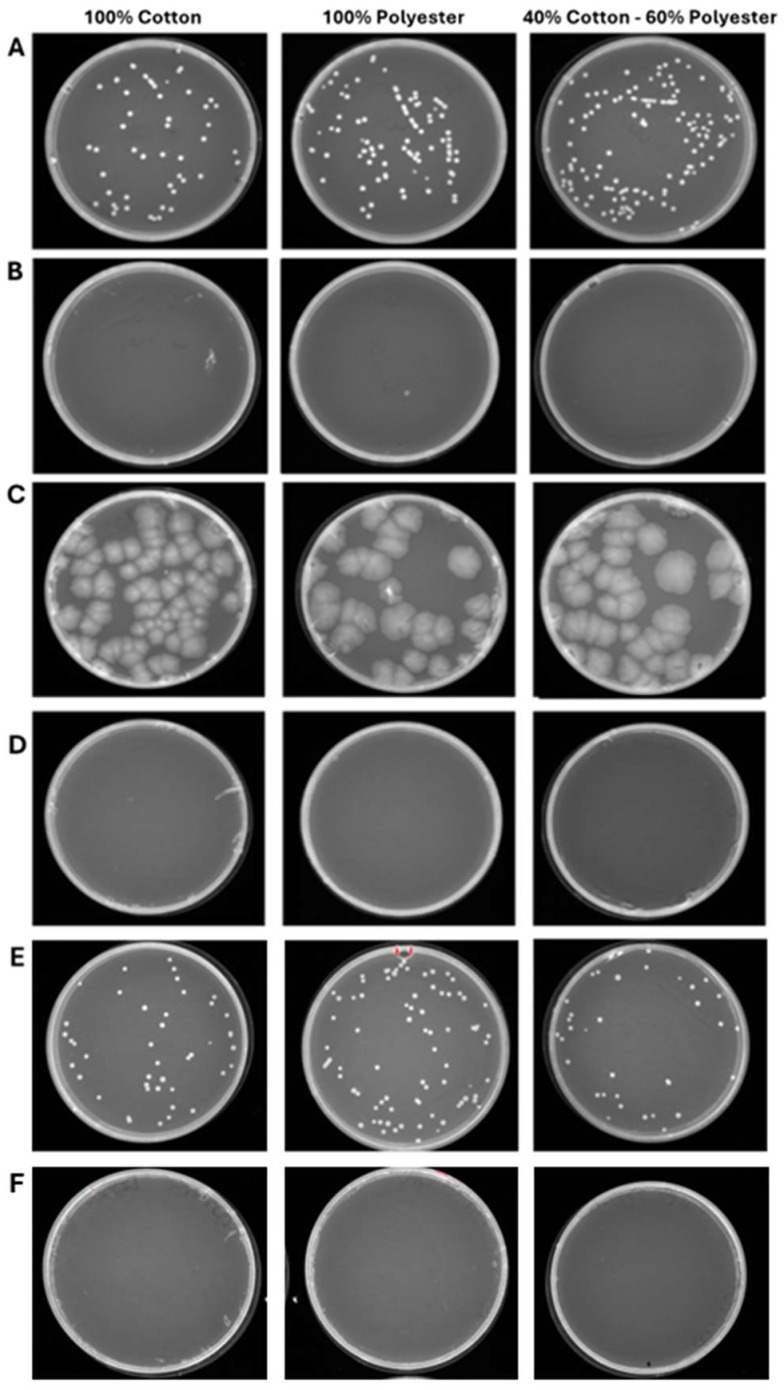
Program 2 (45 min): microbial growth from untreated (**A**) and treated (**B**) *S. aureus*-contaminated fabrics; untreated (**C**) and treated (**D**) *E. coli*-contaminated fabrics; untreated (**E**) and treated (**F**) *C. albicans*-contaminated fabrics. Data represent three independent experiments’ mean ± standard deviation (SD).

**Figure 8:**
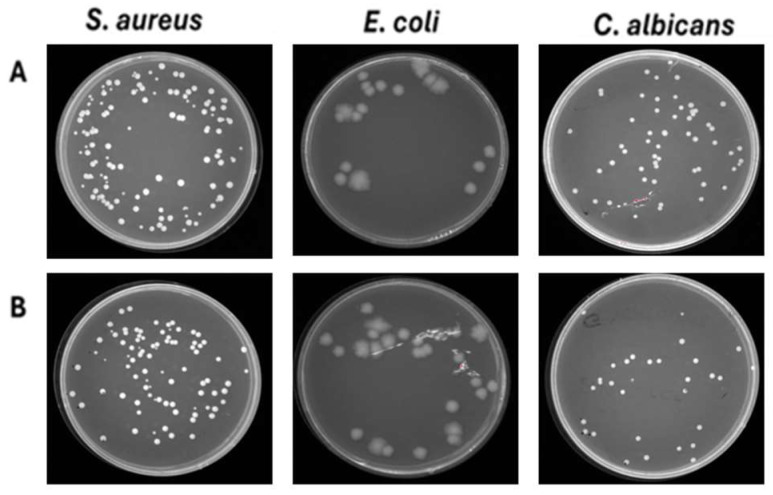
Effect of ventilation against *S. aureus, E. coli and C. albicans* deposited on 40% cotton–60% polyester fabrics. (**A**) Untreated contaminated fabrics. (**B**) Treated contaminated fabrics.

**Figure 9:**
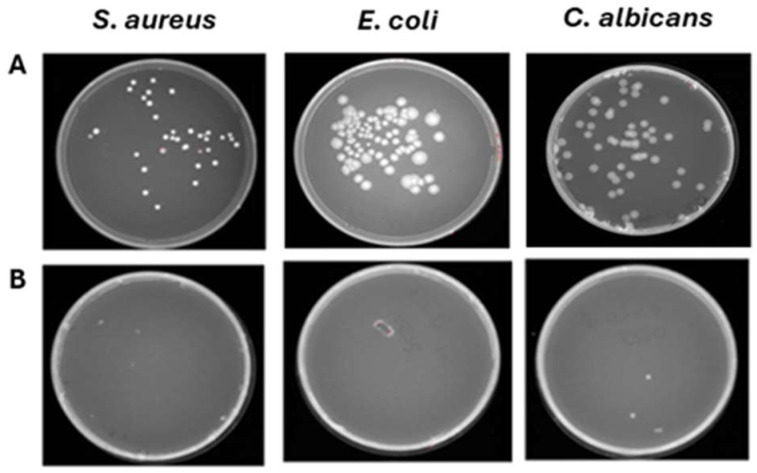
Effect of ozone against *S. aureus*, *E. coli*, and *C. albicans* deposited on 5 layers of 40% cotton–60% polyester fabric. (**A**) Untreated contaminated fabrics. (**B**) Treated contaminated fabrics.

## Error in Acknowledgment Statement

In the original publication, the authors did not acknowledge the use of AI tools for improving the language and readability of the manuscript. The correct Acknowledgement section appears below.

The authors declare that artificial intelligence (ChatGPT) tools were used to improve the clarity and fluency of the English language in this manuscript.

The authors state that the scientific conclusions are unaffected. This correction was approved by the Academic Editor. The original publication has also been updated.
