# Supplementary material for: Optimizing Textile Disinfection in Hospital-Associated Infections Using Gaseous Ozone
Source: Pathogens. 2025 Sep 26;14(10):977. doi: 10.3390/pathogens14100977 (PMC12567330; doi:10.3390/pathogens14100977)
Supplement: Supplementary file 1 [file pathogens-14-00977-s001.zip › pathogens-3831443-supplementary.pdf]

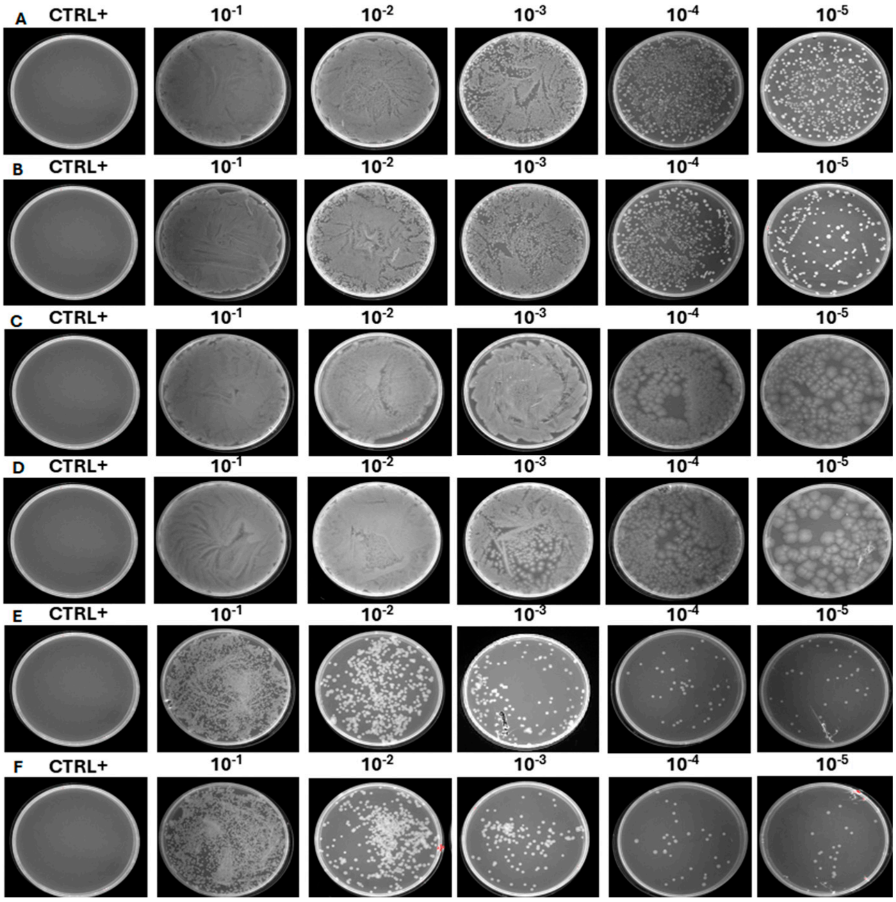

Figure S1. Evaluation of the effectiveness of the compact ozone rotary dispenser on seeded plates with *E. coli*, *S. aureus*, and *C. albicans*. Program 1 (25 min): microbial growth of untreated *S. aureus* (A) and treated with ozone (B); *E. coli* untreated (C) and treated (D); *C. albicans* untreated (E) and treated (F).

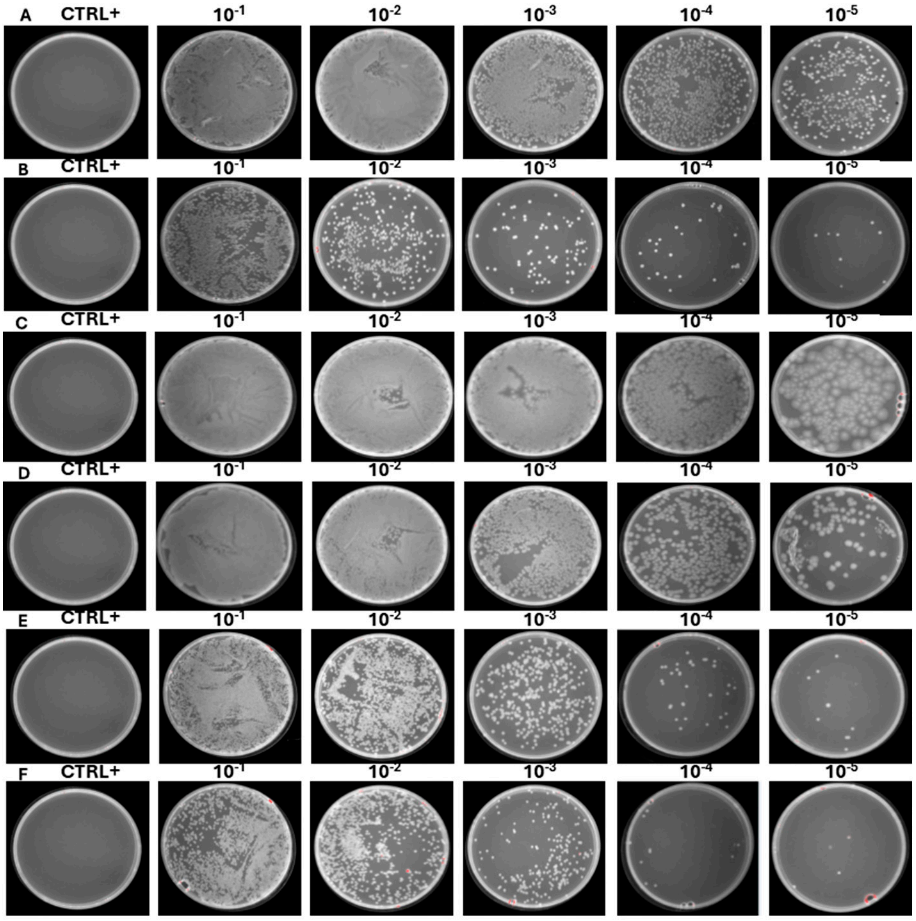

Figure S2. Evaluation of the effectiveness of the compact ozone rotary dispenser on seeded plates with *S. aureus*, *E. coli* and *C. albicans*. Program 2 (45 min): microbial growth of untreated *S. aureus* (A) and treated with ozone (B); *E. coli* untreated (C) and treated (D); *C. albicans* untreated (E) and treated (F).
